# Viral Metagenomic Profiling of Croatian Bat Population Reveals Sample and Habitat Dependent Diversity

**DOI:** 10.3390/v12080891

**Published:** 2020-08-14

**Authors:** Ivana Šimić, Tomaž Mark Zorec, Ivana Lojkić, Nina Krešić, Mario Poljak, Florence Cliquet, Evelyne Picard-Meyer, Marine Wasniewski, Vida Zrnčić, Anđela Ćukušić, Tomislav Bedeković

**Affiliations:** 1 Laboratory for Rabies and General Virology, Department of Virology, Croatian Veterinary Institute, 10000 Zagreb, Croatia; ivanasimzg@gmail.com (I.Š.); nina.lemo@yahoo.com (N.K.); bedekovic@veinst.hr (T.B.); 2 Faculty of Medicine, Institute of Microbiology and Immunology, University of Ljubljana, 1000 Ljubljana, Slovenia; tomaz-mark.zorec@mf.uni-lj.si (T.M.Z.); mario.poljak@mf.uni-lj.si (M.P.); 3 Nancy Laboratory for Rabies and Wildlife, ANSES, 51220 Malzéville, France; florence.cliquet@anses.fr (F.C.); evelyne.picard-meyer@anses.fr (E.P.-M.); marine.wasniewski@anses.fr (M.W.); 4 Croatian Biospeleological Society, 10000 Zagreb, Croatia; vzrncic@gmail.com (V.Z.); andela.cukusic2@gmail.com (A.Ć.)

**Keywords:** bats, viral metagenomics, Croatia, virus, diversity

## Abstract

To date, the microbiome, as well as the virome of the Croatian populations of bats, was unknown. Here, we present the results of the first viral metagenomic analysis of guano, feces and saliva (oral swabs) of seven bat species (*Myotis myotis*, *Miniopterus schreibersii*, *Rhinolophus ferrumequinum*, *Eptesicus serotinus*, *Myotis blythii*, *Myotis nattereri* and *Myotis emarginatus*) conducted in Mediterranean and continental Croatia. Viral nucleic acids were extracted from sample pools, and analyzed using Illumina sequencing. The presence of 63 different viral families representing all seven Baltimore groups were confirmed, most commonly insect viruses likely reflecting the diet of insectivorous bats. Virome compositions of our samples were largely impacted by the sample type: invertebrate-infecting viruses were most frequently found in feces, bacterial viruses in guano, whereas vertebrate-infecting viruses were most common in swabs. Most vertebrate-infecting virus sequences were assigned to retroviruses, parvoviruses, iridoviruses, and poxviruses. We further report the complete genome sequence of a novel adeno-associated virus, densovirus and a near complete length genome sequence of a novel iflavirus. Additionally, one of the most interesting findings in this study was the difference in viromes between two contrasting habitats, the continental and Mediterranean Croatia.

## 1. Introduction

Bats are the second most species-rich taxonomic group of mammals after rodents, representing 20% of mammalian diversity. Part of the *Chiroptera* taxonomical order, these flying mammals inhabit all continents except Antarctica [1]. Bats play an essential role in ecosystems globally and humans benefit from their presence in many ways due to their roles for example in seed dispersal, pollination and their guano being used as an organic fertilizer [1]. Many bats, including all European species, are insectivorous and prey on several insect species that can cause high economic losses [1,2]. Bat populations have been reported to respond to environmental stressors, including habitat and climate alterations, and have been reported as suitable ecological indicators of habitat quality [3]. At least 53 bat species have been identified in Europe [4] and all of them are fully protected by both national [5] and international legislation [6]. Of these, at least 34 bat species are found in Croatia and have been reported to inhabit a wide range of habitats, ranging from forests, underground objects, as well as human settlements [7,8].

It has been estimated that more than 60% of emerging infectious diseases, result from the spillover of pathogens from animal populations, with a majority of these (71.8%) originating in wildlife reservoirs [9]. The emergence of zoonotic viruses, such as coronaviruses (SARS-CoV, MERS-CoV, SARS-CoV-2), filoviruses, henipaviruses or lyssaviruses, with high mortality and transmission rates among humans and livestock, and their association with bats, in recent years, has led to an expansion of research on viruses and their chiropteran hosts [10,11,12,13]. The use of next generation sequencing (NGS) has facilitated the detection of novel bat viruses reported from across the globe. Bat diversity, geographical distribution, biology as well as gregarious behavior are likely important factors contributing to their ability to host a diverse variety of viruses [1,14].

To date, the microbiome, as well as the virome, of the Croatian populations of bats remains unknown. Aside from Heneberg and colleagues [15], who assessed the health-related suitability of some underground sites for military purposes, only a limited number of samples originating from bats have been reported to be tested, and even those only for rabies and white nose syndrome [16,17,18].

The present study is a part of a Croatian nationwide project aimed at the surveillance of rabies and other viral pathogens of zoonotic potential in the Croatian bat population, initiated in 2016. Herein, based on metagenomic analysis of NGS data, bat viromes from guano, feces and oral swabs (saliva) obtained from the several bat species, sampled from different locations in continental and Mediterranean Croatia are reconstructed. The primary aim of this study was to facilitate the assessment of the Croatian bat population as a potential reservoir of viral pathogens with zoonotic potential.

## 2. Materials and Methods

### 2.1. Bats, Locations, Sampling, and Ethics Statement

**Figure 1:**
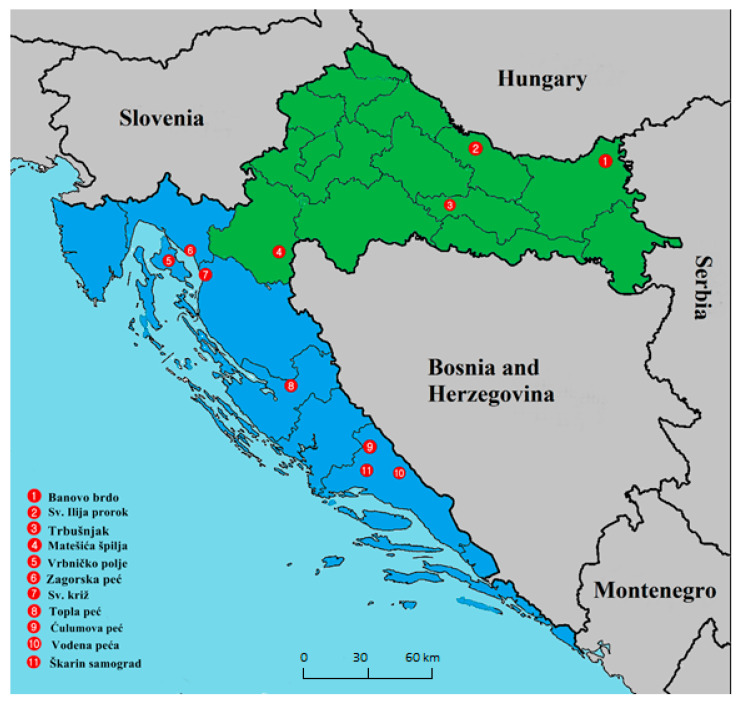
Locations of bat sampling in continental (green) and Mediterranean (blue) Croatia. Source: [19]; permission is granted to copy, distribute and/or modify this map since it is based on free copyright.

Seven species of bats—the greater mouse-eared bat (*Myotis myotis*), lesser mouse-eared bat (*Myotis blythii*), Geoffroy’s bat (*Myotis emarginatus*), Schreiber’s bent-winged bat (*Miniopterus schreibersii*), greater horseshoe bat (*Rhinolophus ferrumequinum*), serotine bat (*Eptesicus serotinus*), and Natterer’s bat (*Myotis nattereri*)—were included in the present study. Bats were sampled during spring 2016, and spring and autumn 2017, at 11 geographical locations in continental (*n* = 4; locations 1–4) and Mediterranean (*n* = 7; locations 5–11) Croatia (Figure 1). The sampled locations were stratified according to presence of human activity in the surrounding landscape between natural locations—locations 2, 7 (churches) and 5 (a tunnel)—and those where the surrounding landscape is more impacted by human activity—location 1 (a closed mine) and locations 3, 4, 6, 8–11 (caves) (Figure 1).

Bats were captured and handled by bat biologists. Captures were facilitated using mist nets (Ecotone Mist Nets, Ecotone, Poland) at the entrances of the caves during night (locations 1, 4, 10 and 11) or using hand nets inside the colony dwellings during the day (locations 2, 3, 5–9). Sampling was repeatedly conducted over the time span of two consecutive years at locations 3, 4 and 11. Recapture of bats at these sites was not evaluated, because the previously captured bats were not marked. During sampling, bats were placed into cotton bags individually and species were determined by bat biologists according to morphological criteria [2]; age, body mass, forearm length, sex, and reproductive status were recorded.

Three types of samples were collected throughout this study. Oral swabs (swabs, i.e., saliva) were collected from individual bats, feces samples were collected from individual bats defecating at the time of sampling, and guano samples were collected from below the colonies where available. Swabs and feces samples were preserved in 500 μL of nucleic acid stabilization reagent (DNA/RNA Shield; Zymo Research, Irvine, CA, USA), already at the time of sampling, whereas guano was only resuspended in phosphate-buffered saline (PBS; 10% wt/vol) immediately before further processing in the laboratory.

All procedures including capture, handling of bats, as well as sample collection, were carried out in accordance with the ethical guidelines and permit delivered by the Croatian State Institute for Nature Protection (consent number: UP/I-612-07/16-48/163). All bats were successfully released at the location of their capture after sample collection.

### 2.2. Sample Preparation and Viral Nucleic Acid Extraction

Oral swabs were pooled according to bat species, sampling date and location, with two exceptions: (i) since only one *Myotis nattereri* was caught it swab was pooled with swabs from *Miniopterus schriebersii* from same location; and (ii), although only one *E. serotinus* was caught, this animal was examined independently because it has been reported as a rabies reservoir previously [20]. In conclusion, there were a maximum of 15 individual swab samples per pool.

All samples were vortexed and centrifuged at 13,000× *g* for 5 min. Consequently, the supernatants were filtered (0.22-µm filters, Millipore, Burlington, NJ, USA) and the filtrates were subjected to nuclease treatment (100 U DNase I, New England Biolabs, Ipswich, MA, USA) at 37 °C for 1 h followed by automatic nucleic acid isolation using iPrepViral Kit and the iPrep instrument (Invitrogen, Carlsbad, CA, USA). Ribosomal RNA was depleted using 30 µL of RNA, 3 µL of Reaction Buffer A, 0.5 µL Riboguard RNase inhibitor and 1 µL Terminator 5′-Phosphate-Dependent Exonuclease (Epicentre Biotechnologies, Madison, WI, USA) as described previously [21]. After the subsequent round of purification using RNA Clean and Concentrator (Zymo Research), the cDNA Synthesis System Kit (Roche Diagnostics, Basel, Switzerland) was used for double-stranded cDNA synthesis, carried out according to the manufacturers’ instructions.

### 2.3. Library Construction and Nextera XT Illumina Sequencing

Double-stranded DNA was quantified using Qubit fluorimeter (Life Technologies, Carlsbad, CA, USA) and diluted to a final concentration at 0.2 ng/µL. Sequencing libraries were prepared using Nextera XT sample preparation kit and the Nextera index kit (Illumina, San Diego, CA, USA) according to manufacturers’ instructions, using 5 µL of diluted dsDNA. Finally, the libraries were sequenced using the 500-cycle MiSeq reagent kit v2 on the MiSeq platform (Illumina).

### 2.4. Viral Metagenomic Profiling

For the purpose of viral metagenomic profiling, the obtained read pairs were merged based on minimum overlap length of 40 nucleotides (nt) displaying a minimum sequence identity of 90%, using CAP3. Merged sequences, longer that 100 nt, were considered in further metagenomic analyses. The sequences were then compared to the GenBank non-redundant protein database (downloaded 21 June 2018 from National Center for Biotechnology Information (NCBI): [22]) using Diamond BLASTx (version GitHub commit: [23,24]), adopting an E-value cut-off of 10^−4^. The Diamond BLASTx search was restricted to the database protein sequences that corresponded to the subset of Viruses (taxid: 10239). The Diamond BLASTx output was used to assign taxonomic classifications to the merged sequences with MEGAN Community Edition (version 6.11.7, built 11 June 2018, [25]). Taxonomic classification output was further processed, summarized and visualized using custom procedural scripts in Bash and Python programming languages, using the functionality provided by numpy, scipy, matplotlib, scikit-learn and pandas python 2.7 modules. Host range information was obtained from the NCBI Taxonomy portal by parsing the html files related to the relevant taxon, using a custom procedural script. It is noteworthy that the lowest level taxon that could be obtained for a given read pair—and that also included a host range description on the NCBI Taxonomy pages—was used, and the information was subsequently summarized upwards until the taxonomical level of family. Data availability: BioProject: PRJNA433098.

### 2.5. Complete Viral Genome Assembly, Identification and Taxonomic Classification

De novo assemblies, aimed to reconstruct complete genome sequences of the viruses in the samples were obtained using SPAdes 3.12.0 [26]. The de novo assembled contigs, longer than 1000 nt, were also searched against the non-redundant protein database, as described above, contigs indicating protein hits were then used in NCBI blastn and NCBI blastx [27] searches to help guide taxonomical classification. Completeness of the de novo-assembled viral genomes was determined by comparing certain characteristics the query contigs and the preliminary taxonomical units assigned to them, including genome length, gene content, sequence similarity (complete and on the gene level), sequence features at the contig flanks, stem-loop signals, etc., as described at the relevant International Committee on Taxonomy of Viruses (ICTV) webpage (https://talk.ictvonline.org/taxonomy/).

### 2.6. Phylogenetic Analyses

Sequence alignments were prepared using Muscle v3.8.31 [28], maximum likelihood phylogenetic trees were constructed using IQ-TREE v1.6.10 [29]. Reliabilities of phylogenetic trees were evaluated using the SH-like approximate likelihood ratio test (SH-aLRT, [30]) with 1000 replicates, the abayes test and the ufbootstrap [31] procedure with 1000 replicates; best fitting phylogenetic models were selected automatically using IQ-TREE functionality [32]. The GeneBank accession numbers of the viral sequences used in phylogenetic analyses are shown on tree figures. Trees were visualized using FigTree (v1.7).

### 2.7. Statistical Analysis

The zeroth- and first-order Hill diversity numbers [33] were calculated as measures of viral diversity, based on the taxonomic and host range classification outputs (denoted: ^0^D_tax_, ^1^D_tax_, ^0^D_host_, ^1^D_host_). While the zeroth order diversity number (^0^D) addresses the number of different taxa identified in each sample, its first-order counterpart (^1^D) introduces a measure of abundance of individual detected taxa into the metric.

Comparisons of numerical variables of multiple groups were carried out using the Kruskal–Wallis test [34] and statistical significance was determined at a threshold *p*-value level of 0.01. Correlation of between pairs of variable vectors was based on the Spearman rank correlation coefficients (ρ).

## 3. Results

### 3.1. Bats, Locations and Sampling

Samples were collected from a total of 455 bats. Most sampled bats were identified as *Miniopterus schreibersii* (*n* = 255), followed by *R. ferrumequinum* (*n* = 90), *Myotis myotis* (*n* = 56), *Myotis emarginatus* (*n* = 10) and *Myotis blythii* (*n* = 27). Only single *E. serotinus* and *Myotis nattereri* each were caught and examined. One bat escaped before its species and sex could be determined and it was not included. Fourteen bats of the *Myotis* genus could not be classified to the species level confidently based on morphological criteria and were excluded from the study as well.

In total, 43 samples, including 28 swab pools (185 swabs from four continental locations and 255 swabs from seven Mediterranean locations), 5 guano, and 10 feces samples were sequenced using NGS. Swabs were collected at all locations (1–11) with the majority collected at locations 3 (*n* = 111) and 11 (*n* = 92). Guano was collected at three Mediterranean (6, 7, 11) and two continental locations (2, 3), whereas fecal samples were collected from three Mediterranean (5, 7, 11) and three continental locations (1, 3, 4) (Figure 1).

### 3.2. Viral Metagenomic Profiling

Details regarding the sample composition of each pool are provided in Table 1. A total of 7,536,096 reads were obtained through sequencing and read pair assembly yielded 1,565,543 merged sequences (termed “sequences”) that were further used for viral metagenomic composition analysis. Of these, 39,527 sequences (2.52%) were attributed to viruses by viral metagenomic profiling (Table 1).

Most sequences were obtained from swab samples (*n* = 1,107,621; 70.75% of total sequences), which represented 65.12% of samples. Only 0.47% (*n* = 5199) of these could be attributed to viruses. Fecal samples displayed both the highest content of viral sequences (50.47%) and contribution to all viral sequences obtained during this study (52.49%) (Table 1). Of all viral sequences, 75.26% and 43.18% identified originated from Mediterranean locations and locations surrounded by natural landscape, respectively (Table 1). The bat species *R. ferrumequinum* displayed the highest number of viral sequences (*n* = 19,060), followed by *Miniopterus schreibersii* (*n* = 1634), *Myotis myotis* (*n* = 955), *E. serotinus* (*n* = 452), *Myotis blythii* (*n* = 380) and *Myotis emarginatus* (*n* = 236). A large number of viral sequences (*n* = 16,810), second in rank, originated from samples that could not be attributed to a single species of bat (Table 1).

### 3.3. Eukaryotic Versus Prokaryotic Viruses

Most viral sequences identified were attributed to eukaryotic viruses (67.13%), while 27.16% of viral sequences represented prokaryotic (bacterial or archaeal) viruses. Invertebrate (83.18%) and vertebrate (12.73%) viruses represented the highest overall percentages of eukaryotic viral sequences, followed by protozoan (3.04%), plant or algal (2.27%), and fungal (0.02%) viruses. A total of 1.02% of viral sequences were attributed to eukaryotic viruses with a wide host range—attributed to more than one of the host range classes described above; 5.71% of viral sequences were attributed to viruses with unknown host ranges (Table 2).

### 3.4. Viral Metagenomic Profiling Revealed Presence of 63 Viral Families; Most Common Were Viruses Infecting Eukaryotes–Insects

The combined dataset indicated traces of 63 families of viruses, belonging to all seven Baltimore groups, including viruses infecting bacteria or archaea, insects, fungi, mammals, and plants or algae, as well as viruses that can infect various eukaryotes and those whose host range remains undefined (Table 1 and Table 2). The most common taxonomical families included the insect-infecting *Iflaviridae* (44.66%, *n* = 17,654; 81.80% of invertebrate infecting viral sequences), followed by *Siphoviridae* (9.59%, *n* = 3791) and *Podoviridae* (7.35%, *n* = 2906). At the same time, *Siphoviridae* and *Podoviridae* represented the majority of bacterial and archaeal virus sequences—35.31% and 27.07%, respectively. A total of 14.24% (*n* = 5628) of viral sequences could not be effectively attributed to any single taxonomical family (Table 2).

*Mimiviridae* and *Phycodnaviridae* were represented by 84.81% and 59.01% of viral sequences among protozoan, and plant and algal viruses, respectively.

Most sequences, representing viruses with a wide eukaryotic host range were attributed to viruses not assigned to any taxonomical family (98.13%), or to the *Peribunyviridae* family (1.87%) (See Table 2 and Supplementary Table S1).

Interestingly, viral sequences related to *Alloherpesviridae* and *Malacoherpesviridae*, respectively, were found at both Mediterranean and continental locations, in oral swabs and guano.

### 3.5. Vertebrate Viruses

Vertebrate virus sequences were mostly comprised of *Retroviridae* (27.89%), *Parvoviridae* (22.65%), *Iridoviridae* (13.14%) and *Poxviridae* (11.51%). Of note, *Reo*-, *Herpes*-, *Flavi*-, *Circo*-, *Astro*-, *Corona*- and *Picornaviridae* each contributed between 1 and 10% of the sequences attributed to vertebrate-infecting viruses, whereas other important vertebrate pathogen viral families such as *Adeno*-, *Calici*-, *Polyoma*-, *Peribunya*-, *Asfaviridae* each contributed less than 1% of sequences attributed to vertebrate-infecting viruses. It should also be noted that among the taxonomical families that are known to contain some of the most noteworthy viruses pathogenic to vertebrate hosts, such as *Polyomaviridae* and *Peribunyaviridae* were only detected in guano samples, *Adenoviridae* only in swab and feces samples, *Coronaviridae* only in swab and guano samples, whereas *Asfaviridae* were only found in swab samples. The presence of *Asfaviridae* was only suggested for a single swab sample pool from location 6. *Coronaviridae* sequences were found in four samples from four different locations (locations: 2, 3, 4 and 11) (Figure 1). We detected traces (five viral contigs) of *Rhabdoviridae* in a single sample: a pool of guano specimens collected the continental location 3. All samples mentioned above in relation to *Asfa*- and *Coronaviridae* originated from locations relatively devoid of human activity. A complete summary of metagenomic assignments is available in Supplementary Table S1.

#### Taxonomical Families of Vertebrate Viruses Interesting Due to Zoonotic Potential

Among 922 retroviral sequence contigs from 36 different samples, there were several species-level taxonomical assignments indicating the presence of human retroviruses, such as human endogenous retroviruses H, K and W, human T-lymphotropic retrovirus, multiple sclerosis-associated retrovirus, human mammary tumor retrovirus, etc., the matching sequences exhibited 45–90% identities at alignment lengths of a mere 30–100 amino acids. There was also a single sequence that, in translation, contained a 30-amino-acid segment with identical to a part of the env protein of human immunodeficiency virus 1 (HIV-1).

The next most abundantly identified group of vertebrate viruses with zoonotic potential were *Parvoviridae*, where we managed to reconstruct two complete genomes of a novel adeno-associated virus and a novel densovirus, and the dataset suggested a further presence of human parvoviruses 4 and B19, with 1 and 2 sequences, respectively, all alignments indicated between 60% and 64% similarity to the database protein sequences and at alignment lengths between 34 and 36.

Poxviral sequences included various species-level taxa including entomo- as well as chordipoxviruses, such as Canary-, Fowl-, and Pidgeonpox viruses, Deerpox virus, NY_014, Murmansk and Yokapox, and Tanapox viruses, viruses Vaccinia and Ectromelia, Cowpox, Mythimna separata entomopoxvirus, Orf virus, Molluscum contagiosum virus and others, all at short alignment lengths at various levels of similarity.

Among *Reoviridae* we found indices of Rotaviruses A, B, C, F, G and others, including several of their taxonomical subunits. Most interesting were the sequences indicating the presence of Human rotavirus A, highlighted by the presence of sequences representing several of its genes (VP2, VP3, VP6, VP8, NS3, NS5), at similarity values ranging from 45–100%. All reovirus sequences were found in continental locations 1, 2, 3 and 4, surrounded both, by natural and anthropogenic landscape, in swab, feces and guano samples.

Among *Herpesviridae* we found indications of presence of Human alphaherpesvrus 1 (Human herpesvirus 1–HHV-1/Herpes simplex virus–HSV; 44 sequences), Human betaherpesvirus 5 (human cytomegalovirus–HCMV; 3 sequences) and Human gammaherpesviruses 4 and 8 (Epstein-Barr virus–EBV and Kaposi’s sarcoma-associated herpesvirus–KSHV; 3 and 2 sequences), in nine, three and one different samples, respectively. KSHV alignments were 65 and 46 amino acids long with 34% and 50% identity to the reference protein sequences; they did not indicate any specific protein function. EBV sequences indicated variable alignment similarities of 58%, 74% and 45% at alignment lengths of 69, 98 and 95 amino acids, and indicated relatedness to a ribonucleotide reductase, a viral Stanniocalcin analog and a protein without a known function, respectively. Sequences resembling HCMV aligned at relatively low similarities of 35%, 50% and 53% at alignment lengths of 128, 45 and 80, respectively. While the first of the three sequences aligned to a protein without a specific function, the latter two hinted relatedness to a viral chemokine family protein. All HHV-1-like sequences resembled a viral glycoside hydrolase enzyme at variable similarity rates, ranging from 35 to 100%. In all of the above cases, we would speculate that she sequences either originated from a similar herpesvirus or actually represent host contaminants. This is due to the fact that (i), in all cases, only a single protein (or proteins from a single focal point in the viral genome) was detected, as well as (ii) the mentioned low sequence similarities at the limited alignment lengths.

*Adenoviridae*-like sequences resembled mainly different bat, porcine and canine mastadenoviruses. A single sequence indicated similarity to the Pre-hexon linking protein IIIa of Human mastadenovirus F, although at similarity level of 75% and at the alignment length of 52 amino acids.

*Coronaviridae*-like sequences included representatives of both alpha and betacoronaviruses, but mainly limited to bats in terms of host ranges. A single sequence indicated peak protein sequence similarity to short stretch of the spike glycoprotein of severe acute respiratory coronavirus (SARS-CoV) found in bats in Yunnan province, China in 2016. Although the alignment exhibited 91% identity, the sequence stretch spanned a mere 32 amino acids, and could have actually originated from another, similar, but so far unknown, betacoronavirus.

We did not identify any potential human pathogens among *Flaviviridae*-like sequences, these resembled mainly Pestiviruses A and B, one sequence resembled a protein from Hepatitis virus GB type B. Among *Peribunya*-, *Astro*- and *Caliciviridae*-like sequences, we found indication of only bat virus-like sequence, while *Picornaviridae* several sequences resembling insect-infecting viruses, with no viruses with potentially harmful to humans. All five *Rhabdoviridae*-like sequences resembled plant rhabdoviruses and none indicated presence of mammalian rhabdoviruses. Presence of African swine fever 1 might have been suggested by the detection of the single *Asfaviridae* sequence and the single *Polyomaviridae* sequence indicated presence of an arachnid polyomavirus.

### 3.6. Three Complete and One Nearly Complete Genome Sequences of Novel Viruses Were Identified

After de novo assembly of read pairs we identified and characterized three complete novel genome sequences, including an adeno-associated virus (Adeno-associated virus Croatia cul1_12; GenBank Acc. No.: MN099037), a densovirus (Ambidensovirus Croatia 17_S17; GenBank Acc. No.: MN099038) and of a novel circo-like virus (Circo-like virus Croatia 17_S17; GenBank Acc. No.: MK241555). Furthermore, two partial, nearly complete, genome sequence contigs (Iflavirus sp. strain 15/G-Me polyprotein gene and Iflavirus sp. strain 16/F-Rf polyprotein gene; GenBank Acc. Nos.: MG963177, MG963178), that may originate from divergent viruses of the *Picornaviriales* order, likely from the *Iflaviridae* family, were identified. The complete genome sequence Circo-like virus Croatia 17_S17 has been described previously in a separate publication [35]; most notably, it was found to display peak similarity scores to viruses found in human-derived samples.

#### 3.6.1. Iflavirus

Two sequence contigs, likely originating from viruses of the family *Iflaviridae* (order *Picornavirales*), 4201 and 1864 nt in length, respectively, were found in guano collected from under mixed colonies of *Myotis emerginatus* and *R. ferrumequinum* and from the individual feces of *R. ferrumequinum* at location 7. Sequences were generated from 1158 sequencing reads with mean coverage = 93 (GenBank accession No.: MG963177, MG963178). The two-nucleotide sequence contigs assembled de novo displayed 74% and 78% NCBI blastn identities, to *Spodoptera exigua* iflavirus 2 isolates from Spain (GenBank Acc. No.: KJ186788) and Korea (GenBank Acc. No.: JN870848), respectively.

#### 3.6.2. Ambidensovirus

**Figure 2:**
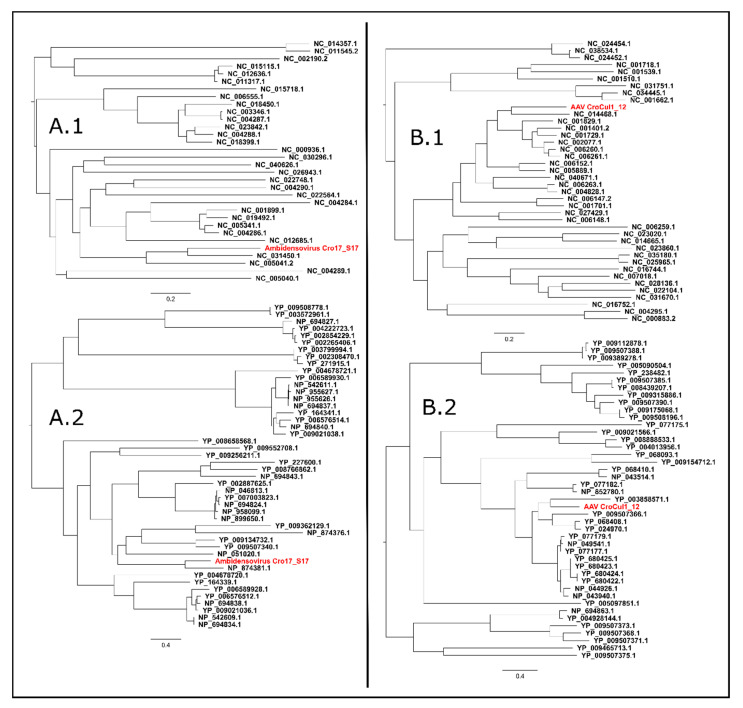
Phylogenetic clustering of Ambidensovirus Cro17_S17 (**A**) and Adeno-associated virus AAV CroCul1_12 (**B**) based on the complete genome nucleotide (**A.1**,**B.1**) and the NS1/Rep amino acid contexts. All phylogenetic trees were inferred using IQ-TREE, best-fitting phylogenetic models were selected automatically based on the Bayesian information criterion; all trees were visualized using FigTree and rooted at mid-point. The complete genome phylogenetic trees were constructed based on the complete genome sequences matching *Densovirinae* (**A.1**) and *Parvovirinae* (**B.1**) taxons in NCBI RefSeq using phylogenetic models GTR+F+R4 and GTR+F+R6, respectively. The NS1/Rep protein phylogenetic trees were constructed based on NS1 and Rep proteins sequences from the NCBI Non-redundant protein dataset matching *Densovirinae* (**A.2**) and *Parvovirinae* (**B.2**) taxons, using phylogenetic models VT+F+R4 and VT+F+R9.

A complete genome sequence of a novel virus, named Ambidensovirus Croatia 17_S17, exhibiting peak NCBI blastn identities to denso and denso-like virus sequences in GenBank, was identified in a sample of guano collected under the colony of *Miniopterus schreibersii* at location 6. The complete genome sequence of the newly identified virus was 6110 nt long, it contained short 3′- (211 nt) and 5′- (287 nt) terminal palindromic sequences and contained five open reading frames (ORFs). The identified ORFs putatively encode three parvoviridal non-structural proteins (NS1, NS2 and NS3) in the 5′ terminal region of the positive DNA strand, and two structural proteins (VP1 and VP4) in the 5′ terminal region of the negative DNA strand. The complete nucleotide genome sequence was covered on average 71.48× by 3126 sequencing reads and indicated peak nucleotide sequence identity values of 70.6% and 64.2% to the NCBI RefSeq complete genome sequences NC_031450 (*Parus major* densovirus isolate PmDNVJL, complete genome) and NC_005041 (*Blattella germanica* densovirus 1, complete genome), respectively, as determined by sequence demarcation toolkit (SDT; v1.2; [36]). The NS1 protein sequence of the novel virus displayed a peak amino acid sequence identity of 60.15% to sequence NP_874381, the *Blatella germanica* densovirus 1 NS1 protein. Phylogenetic clusterings, embedding all available Densovirinae NS1, Rep1, Rep68 and Rep78 proteins (obtained from the non-redundant protein dataset), and complete genome sequences (obtained from RefSeq) in NCBI, indicated monophyletic relationships of the novel virus with the Ambidensovirus clade (Figure 2A).

#### 3.6.3. Adeno-Associated Virus (AAV)

A complete genome sequence of a novel adeno-associated virus, named Adeno-associated virus (AAV) Croatia cul1_12, NCBI blastn identities to adeno-associated virus sequences in GenBank, was found in oral swabs collected from *R. ferrumequinum* at location 9. The complete genome sequence of the novel AAV was 4561 nt long and indicated the presence of two ORFs, encoding a putative replicase (Rep; in the 5′-terminal region) and a putative capsid protein (Cap, in the 3′-terminal region); both ORFs were found on the same DNA strand. The complete genome sequence was flanked by 38 nt long inverted terminal repeat (ITR) regions on the 5′ and 3′ termini, the complete sequence was covered in average 91.27×, by 4117 sequencing reads. Phylogenetic clustering and nucleotide and amino acid identity values were estimated by embedding the complete genome sequences (RefSeq) and NS and Rep sequences (Non-redundant protein dataset) in NCBI, corresponding to the taxonomical sub-family *Parvovirinae*, and indicated a peak nucleotide sequence identity of 68.8% to the RefSeq sequence NC_014468 (Bat Adeno-associated virus YNM, complete genome) and a 70.5% peak amino acid identity value in the replicase protein to sequence YP_680424.1, representing the Rep40 protein of Adeno-associated Virus 2. Both the complete genome and the replicase protein of the novel virus clustered monophyletically alongside dependoparvoviruses (Figure 2B).

### 3.7. Quantitative Analysis of Diversity and the Viral Compositions of Sample Groups

**Figure 3:**
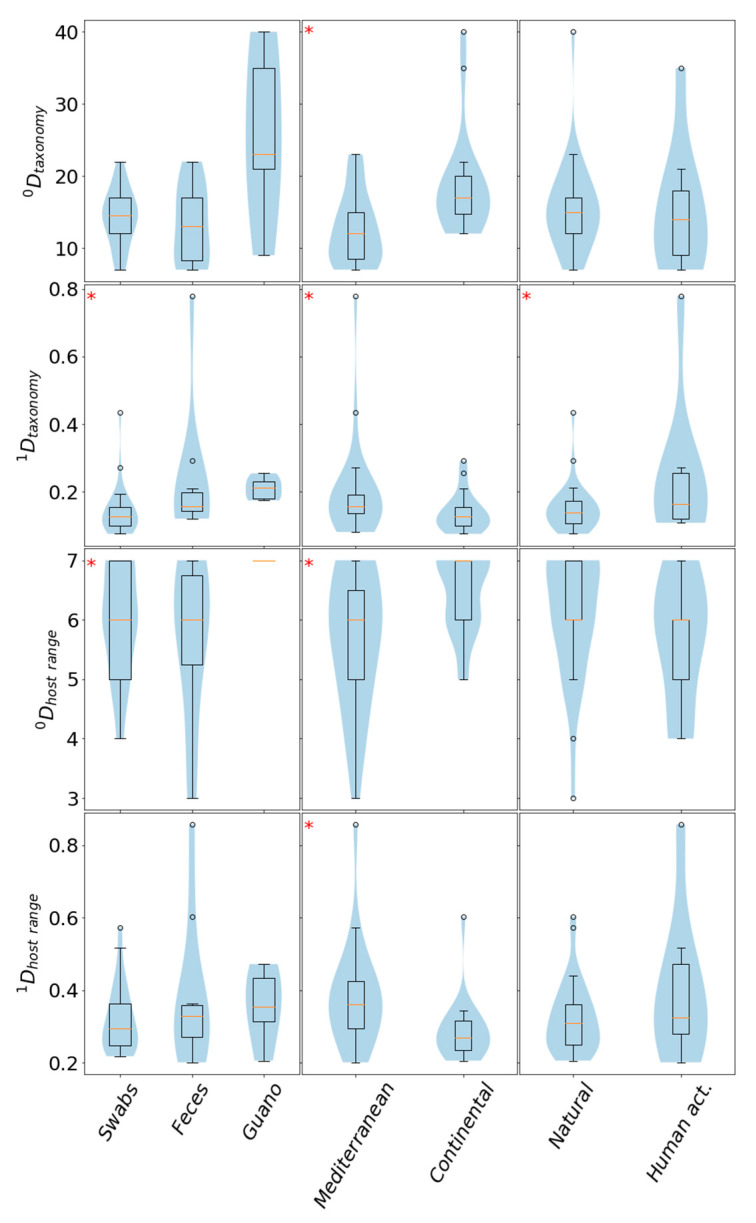
Taxonomy and host-range-based Hill diversity indices (D) calculated by grouping samples according to sample type (oral swab, feces, guano), geographical locations from where the samples were obtained (Mediterranean, continental) and whether the surrounding landscape is subject to human activity (Natural, Human act.). * denotes there may be statistically significant differences (*p* < 0.01) between the population medians of the strata, based on the Kruskal–Wallis test. The diversity indices are depicted a violin plots, widths of the “violins” correspond to the kernel density estimates at the given ordinate, and as box and whisker plots, indicating the data mean (orange line), the box stretches between the first (Q1) and the third quartile (Q3), and the whiskers enveloping the most extreme values lower than Q1 or higher than Q3 by more than 1.5 * (Q3-Q1); values beyond the whisker definition are depicted as individual circles/points.

Viral diversities in samples were estimated by calculating the zeroth- (number/count of different categories in sample/group) and first-order (normalized count expressing deviation from uniformity of representation) Hill diversity numbers, both in the context of individual samples as well as based on data, grouped according to different attributes: geographical location, landscape type, sample type and bat species (Table 1 and Table 3, Figure 3). In feces, guano and swabs we identified 30, 56 and 39 different viral families, respectively (Table 1). Grouped according to bat species, we found most viral families in samples obtained from *R. ferrumequinum*, followed by *Miniopterus schreibersii*, *Myotis myotis*, *E. serotinus*, *Myotis emarginatus* and *Myotis blythii* (Table 1). We found more different taxonomical families in samples originating from locations of natural landscape, compared to those affected by human activity (Table 1). Similarly, continental locations exhibited higher numbers of different viral families than Mediterranean ones (Table 1).

It should be noted that Table 1 lists the diversity indices respective of the various groupings, as calculated directly from the sets of different categories identified (and their relative abundances) in the selected segment of the data, while Figure 3 intends to visualize the spreads of the diversity indices calculated from individual samples (Table 3) belonging to the relevant groups. In contrast to the somewhat drastic differences between the taxonomical ^0^D indices in Table 1, in the case of grouping according to sample type no statistical significance could be identified (*p* < 0.05, [34]) in the sample-based groupwise comparison of taxonomical ^0^D indices (Figure 3). On the other hand, the taxonomical ^1^D and the host range ^0^D indices also indicated statistically significant discrepancies (*p* < 0.05 [34]). In further detail, in the context of ^1^D_tax_, both feces and guano samples appeared to differ from swab samples, whereas in the context of ^0^D_host range_ swabs and feces differed from guano samples (*p* < 0.05; Mann–Whitney U test). Samples from continental Croatia displayed significantly higher numbers of both unique taxonomical families as well as host-range categories (^0^D, Figure 3). On the other hand, Mediterranean samples displayed significantly higher ^1^D diversities, suggesting higher evenness of the represented categories (Figure 3). This seems reasonable since only 9780 viral sequences were obtained from continental but 29,747 from Mediterranean locations and could suggest an insufficient sampling depth of continental locations. Importantly, significant differences could also be observed between the medians of sample ^0^D_tax_, ^1^D_tax_, ^0^D_host_ indices following grouping according to sample type (Figure 3).

Analysis of Spearman rank correlation coefficients (ρ) indicated weak positive correlations might be present between the number of viral sequences and the values of the ^0^D indices in the dataset. The ρ(^0^D_tax_) and ρ(^0^D_host_) amounted to 0.585 and 0.558, with empirical probabilities for uncorrelated systems of 3.84 × 10^−5^ and 1.02 × 10^−4^, respectively, although these probability values may not be reliable at the tested sample size (N_samples_ = 43). A complete/representative sampling of given a population could be considered as one, where the addition of new data regarding that same population does not change the end result (information) significantly—in other words, a steady state, where all variation can be attributed to random dispersion, that is, a system where no correlation would be observed for the abovedescribed statistical setup.

**Figure 4:**
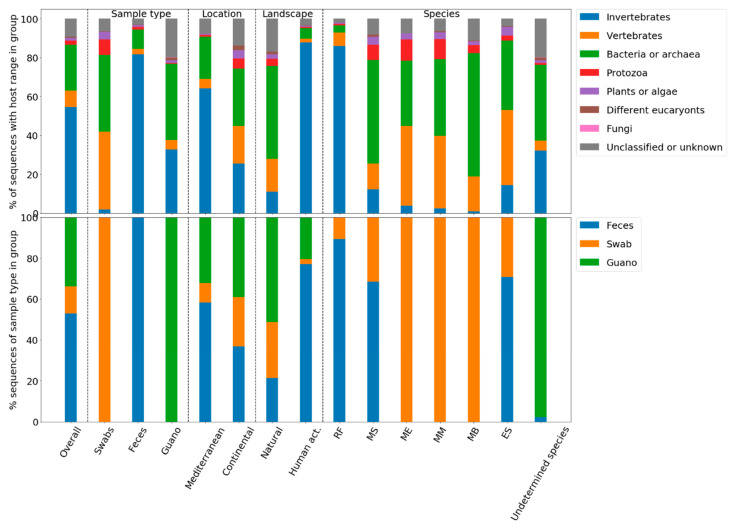
Compositions of samples according host range of classified viral sequences. Samples were grouped according to different attributes (left to right): overall, sample type, location, natural/anthropogenic landscape, bat species.

Most obviously, the present data suggested that the viral composition in a given sample group could to a large degree be explained by the proportion of viral sequences derived from one of the three sample types, namely swab, feces or guano (Figure 4). Feces samples contained the highest proportion of invertebrate viruses; guano samples the highest proportion of bacterial and archaeal viruses, whereas vertebrate infecting viruses were most commonly represented in swab samples. Figure 3 clearly demonstrates that proportions of viral sequences from a given sample type play a crucial role in the host-range-based composition.

## 4. Discussion

Bats represent an important reservoir of emerging and re-emerging viral diseases for humans and animals [37]. Using metagenomic analysis based on NGS, the frequency of new bat virus discovery has increased drastically in recent years [38]. Herein, the first insights into the virome of the Croatian bat population have been revealed. We examined the viral metagenomes in oral swabs, feces and guano of Croatian bats.

In the present study, 2.52% (39,527/1,565,543) (Table 1) of sequences exhibited similarity to known viruses. Slightly lower values have been reported previously in the context of similar studies, such as 1.62% in Myanmar [38] and 0.92% overall in China [39].

We identified a total of 63 viral families (Table 1), while a total of 24 [38] and 51 [40] taxonomical families of viruses have been reported previously in Myanmar and French Guiana, respectively. We considered species level taxonomy assignments as unreliable, and they were rather summarized to the level of taxonomical families, as the minimum nucleotide sequence length of 100 nt (and 33, translated to amino acid code) does not offer sufficient species level specificity and the metagenomic outputs should be interpreted with extreme caution. We consider identification of a given viral taxonomical family in the metagenomic output as indication of presence, whereas only assembly of complete genome or partially assembled genome sequences can serve as firm evidence for the presence of a given virus.

Eukaryotic viruses predominated with 67.13% of viral sequences while prokaryotic (bacterial and archaeal) viruses were represented by 27.16% of viral sequences which is similar to the findings of He et al. (73.5% eukaryotic viruses and 26.5% prokaryotic viruses) [38]. Among prokaryotic viruses, *Siphoviridae* and *Podoviridae* were the most commonly identified viral families (Table 2), which is in line with results of previous studies: *Siphoviridae* and *Podoviridae* were the two most commonly identified prokaryote-infecting viral families in He et al. [38] and Salmier et al. [40].

The largest proportion of viral sequences related to eukaryotic viruses (83.18%) was attributed to viruses infecting invertebrates. With the exception of the Egyptian Fruit bat *Rousettus aegyptiacus* which feeds on fruit, all known European species of bats are insectivorous [1,2], and it is likely that the high relative abundance of insect-infecting viral sequences reflects the bats’ diet, highlighting the bat’s bio-insecticidal role in the environment. It should also be noted that the highest relative abundance of invertebrate-infecting viral sequences was found in feces, reinforcing the idea of their “alimentary” origin. The notion of the bats’ bio-insecticidal role in Croatia was further reinforced by the identification of two partial genome sequences, assembled de novo during the present study from sequencing reads, of a currently unknown virus likely belonging to the *Iflaviridae* family, a family of insect viruses. The two sequence contigs were found in guano and feces collected at a Mediterranean location 7 and most closely resembled sequences of *Spodoptera exigua* iflavirus 2 from Spain and Korea. *Spodoptera exigua* is a species of moth, a common and well-known agricultural pest infesting different vegetables commonly grown in Croatia [41]. *Iflaviridae* were also the most commonly identified viral family in this study, as well as in samples from Mediterranean Croatia.

We also identified a complete genome sequence of a novel ambidensovirus, of the *Parvoviridae* family. The novel ambidensovirus indicated peak similarities to an ambidensovirus identified from the bird great tit (*Parus major*) [42] and the German cockroach (*Blattella germanica*) [43]; however, it should be classified as a novel species in the *Ambidensovirus* genus, according to current ICTV taxonomic classification criteria [44]. The closely related densovirius, detected by Yang and colleagues [42], was originally found in the lung tissue of a great tit, however, they were unable to determine whether the bird was infected by the densovirus or if the densovirus come from insects ingested by the bird without infection of the avian cells [42]. The fact that the ambidensovirus was detected in guano and was also highly similar to a densovirus identified in the German cockroach [43] indicates an alimentary origin of the virus.

An AAV, another member of the *Parvoviridae* family, subfamily *Parvovirinae*, genus *Dependoparvovirus*, was also identified. Adeno-associated viruses have up to now been recorded in bats from China, the USA and Myanmar [45], and, according to our best knowledge, this is the first evidence of an AAV in European bats. Additionally, this is first record of AAV in bat oral swab while in other research it was found in feces [45]. Although AAV usually requires co-infection with helper adenovirus or herpesvirus [46] none of these viruses could be found in the respective swab sample. According to current ICTV taxonomic classification criteria [47], the novel virus should be classified as a novel species in the *Dependoparvovirus* genus.

A novel circo-like virus, was identified in this study, circo-like virus Croatia 17_S17 (Circo-like virus Croatia 17_S17, GenBank Acc. No.: MK241555) has been described by our research group previously in the context of a genome announcement [35]. It indicated a similarity to Circo-like viruses Brazil HS1 and HS2, which were identified in human feces [48,49].

Sequences related to vertebrate viruses made up 12.73% of the bat virome, similar to reports from North America (<10%) [50,51], but contrasts the 45.2% vertebrate infecting viruses in Myanmar [38]. The observed difference could be attributed to the sample types used: studies where a lower percentage of sequences were related to vertebrate viruses, including our predominantly used feces, oral and/or fecal swabs as samples, while He et al. [38] used actual bat organ tissues. Furthermore, our results suggested a strong influence of sample type over the identified virome, as differences in both diversity and compositions of samples originating from different tissues. Feces contained the highest proportion of invertebrate viruses, guano bacterial and archaeal viruses, whereas vertebrate infecting viruses were most commonly represented in swabs. The highest relative abundance of invertebrate viruses in feces likely reflects the diet of bats while the predominance of prokaryotic viruses in guano is probably consequence of its bacterial colonization and decomposition. Considering that some of the viruses may not be excreted through fecal and oral routes, and some of them may have intermittent excretion, feces and oral swabs might not reveal the complete viromes [38]. However, due to the protected status of European bats, the chosen samples were only available.

Although swabs were the most numerous sample types (65.12% of samples, 70.75% of reads) in this study, they contributed only 13.15% viral sequences. On the other hand, despite the highest content of viral sequences and overall contribution (50.47%) to all viral sequences, feces exhibited the lowest viral diversity (^0^D_tax_ = 30), and the least uniformly distributed representation of the identified viral families (the highest ^1^D_tax_/^0^D_tax_ = 0.015). In contrast, guano had the highest viral diversity (56 viral families), both guano and swab samples indicated comparable ^1^D_tax_/^0^D_tax_ values (0.0021—swab; 0.0018—guano), indicated similar evenness of the relative abundances of the identified taxonomical families.

One of the most interesting findings in this study was the difference in viromes between two contrasting habitats, the continental and Mediterranean regions of Croatia. Most viral sequences came from Mediterranean habitats, but it was habitats from continental Croatia that contributed the most sequences in total (Table 1). On the other hand, even though the majority of viral sequences were detected at Mediterranean locations, the higher viral diversity was actually found in continental Croatia. This finding was primarily due to the presence of diverse plant viral families such as *Betaflexviridae*, *Bromoviridae*, *Luteoviridae*, *Solemoviridae*, *Tombusviridae*, *Tymoviridae*, and *Virgaviridae*. These plant viral families probably reflect the plant-based diet of the insects included in the bats alimentary chain. Additionally, when this finding is put in a geographical context, the majority of continental sampling sites were located at the Pannonian basin, one of the major European agricultural areas [52], this would further solidify the aspect of the bio-insecticidal role of bats, as well as their role as a bioindicator in the Croatian ecosystem. Viewed through the lens of evenness according to the ^1^D_tax_/^0^D_tax_ ratio, continental Croatia exhibited a more uniform distribution of the identified viral families in comparison to Mediterranean Croatia. It is generally assumed that a disturbance into an ecological system, such as the heavy agricultural exploitation of the Pannonian basin, would diminish the diversity and evenness of its occupants [53,54], thereby reducing the system’s ability to adapt to change, leading to relative extinction of the majority and the proliferation of some species. In the present case, we would have expected a higher taxonomical evenness—a lower ^1^D_tax_/^0^D_tax_ ratio—in continental compared to Mediterranean Croatia. On the other hand, cross-referencing the continental/Mediterranean sample grouping with grouping based on landscape type revealed that while approximately one-third of Mediterranean locations were considered as anthropogenic (7/23) while only one-sixth of such samples came from continental Croatia. It was also of interest that the mentioned grouping, according to human activity in the surrounding landscape, suggested both a higher species richness in natural locations (^0^D_tax_: 61 vs. 45) as well as higher evenness (^1^D_tax_/^0^D_tax_ ratio: 0.0013 vs. 0.0095).

Regarding bat species, the highest number of viral sequences and highest viral diversity was found in *R. ferrumequinum* and *Miniopterus schreibersii*, which is likely also a consequence of unbalanced sampling, as these bat species were the most represented in the samplings.

Among the raw metagenomic output, we did detect clues suggesting the presence of potential human pathogens, specifically among sequences resembling *Retro-*, *Pox-* and *Herpesvidiae*. It is interesting that, in most cases, poxvirus-like sequences indicated similarity to different viral enzymes or receptors, such as the Molluscum contagiosum virus glutathione peroxidase, Orf virus analog of a type 2 taste receptor, a vaccinia virus analog of a glycoside hydrolase enzyme, the ectromelial complement control protein C3/B5, etc., all viral proteins involved in the interplay with the host organisms and immune systems, which per se also indicate high similarities to their respective mammalian analogues. It may be that all these viral proteins, and by consequence the virus species in question were present in the samples, but it is also possible that the sequences came from the protein’s mammalian analogues. *Retroviridae* on the other hand have coexisted with some of their mammalian hosts for very long evolutionary periods and may exist in their hosts genomes in the form of endogenous retroviruses [55,56,57,58], and in some cases the delineation between host and viral DNA may have faded through away through time. Although it is possible that all these retroviruses were present in the bat samples these species-level taxonomy calls we considered as less reliable as they may also indicate presence of viruses similar to those detected (i.e., from the same governing taxonomical unit, such as family), but in the specific cases, mentioned above, may also have been misinterpreted and actually originated from the hosts DNA. From the same aspect of curation, these results may suggest, that the relative abundances of *Retro-*, *Pox-* and *Herpesviridae* may have been overestimated in this study. On the other hand, the fact that we identified a large portion of reoviral genes in our samples, we do not doubt their presence, it should however be noted (again) that, due to low similarity values it is more likely that the sequences originated from different unknown rotaviruses, and not specifically human rotavirus A.

On a note of caution, the observed correlation between the number of viral sequences and the diversity indices could be interpreted to suggest that more viral families would be identified if the sequencings were augmented in depth. Also, as Li and colleagues [50] stated viral metagenomics can provide relevant sequence data on the most prevalent viruses present in samples but underestimate the diversity of low-concentration viruses. Sequencing depth is thus one of the most important factors to consider in the design of future viral metagenomics studies.

In this study, approximately 14% of viral sequences could not be assigned to any known viral family, which is a lower rate in comparison to similar research [50,54] and could be due to growth of publicly available reference genome databases during last year’s. In the future it could be expected further decrease in this portion [59].

Finally, great care should be taken when interpreting the results of metagenomic studies, especially when it comes to an understudied field such as viromics. Although metagenomics can be a great investigative tool to nonspecifically uncover the viromes and metagenomes of various samples, it is in the same manner prone to the error. As there is no reference genomes for all viruses it is impossible to exclude that some sequences were classified inaccurately, i.e., errors may simply arise from unspecific sequence mappings. This was supported by finding parts of genomes of some viral families which are not typical for bats such as *Alloherpesviridae* and *Malacoherpesviridae*, known to normally infect fishes and molluscs.

## 5. Conclusions

The viral metagenomics data presented in this study provide a preliminary view of virome in Croatian bat population. The primary aim of this study was to facilitate the assessment of the Croatian bat population as a potential reservoir of viral pathogens with zoonotic potential. The further characterization of the bat virome will increase our understanding of mammalian virus diversity and timely detection of potential human pathogens. This study contributes significantly to better understanding of global diversity of bat viruses. Most importantly, in order to compare viral populations in bats from different parts of the world or of different species, effectively–factors such as sample type, habitat and sequencing depth are paramount and should be considered and evaluated with utmost rigor. Also, presence of adequate reference databases play crucial roles in the definitions of viral sample compositions, and further interpretations related to downstream analysis.

## Figures and Tables

**Table 1:** Summary the sample data, stratified location groups (M—Mediterranean, C—continental), sample types, types of landscape, with total numbers of sequences, the numbers of viral sequences and the relevant proportions, and the zeroth- and first-order Hill diversity indices (^0,1^D) based on taxonomy and host range. RF—*R. ferrumequinum*, MS—*M. schreibersii*, MM—*M. myotis*, ME—*M. emarginatus*, MB—*M. blythii*, ES—*E. serotinus*.

Category	All Sequences	% of Total Sequences	Viral Sequences	% Viral Sequences in Category	% of Total Viral Sequences	No. _Samples_	% of _Samples_	^0^D_Tax. Family_	^1^D_Tax. Family_	^0^D_Host Range_	^1^D_Host Range_
**Sample type**											
feces	171,700	10.97	19,951	11.62	50.47	10	18.61	30	0.457	6	0.558
guano	286,222	18.28	14,377	5.02	36.37	5	16.28	56	0.103	7	0.346
swab	1,107,621	70.75	5199	0.47	13.15	28	65.12	39	0.080	6	0.312
**Location type**											
M	428,175	27.35	29,747	6.95	75.26	23	53.49	45	0.243	7	0.427
C	1,137,368	72.65	9780	0.86	24.74	20	46.51	58	0.055	6	0.227
**Landscape type**											
natural	1,242,052	79.33	17,066	1.37	43.18	34	79.07	61	0.081	7	0.309
with human activity	323,491	20.66	22,461	6.94	56.82	9	20.93	45	0.428	6	0.65
**Species**											
RF	449,808	28.73	19,060	4.24	48.22	11	25.58	35	0.490	6	0.605
MS	347,268	22.18	1634	0.47	4.13	12	27.91	29	0.089	6	0.317
MM	267,273	17.07	955	0.36	2.42	4	9.30	23	0.088	6	0.294
ME	35,720	2.28	236	0.66	0.60	1	2.33	17	0.122	5	0.292
MB	36,312	2.32	380	1.05	0.96	3	6.98	13	0.164	6	0.444
ES	79,587	5.08	452	0.57	1.14	2	4.65	18	0.119	6	0.279
Undetermined, other	349,575	22.33	16,810	4.81	42.53	10	23.26	58	0.093	7	0.327
**Total/Overall**	**1,565,543**	**100.00**	**39,527**	**2.52**	**100.00**	**43**	**100.00**	**63**	**0.149**	**7**	**0.342**

**Table 2:** Taxonomical (family level) compositions of the studied samples grouped according to various attributes. Table also denotes the Baltimore classifiers corresponding to each taxonomical family taxon and the host ranges, which were identified for the viral sequences contributing to the taxonomical family composite, based on the data in NCBI. The data are stratified according to sample type (S—oral swab, F—feces, G—guano), geographical location (M—Mediterranean, C—continental), landscape type (N—natural, H—with human activity/anthropogenic) and according to bat species (MS—*M. schreibersii*, MM—*M. myotis*, RF—*R. ferrumequinum*, ME—*M. emarginatus*, ES—*E. serotinus*, MB—*M. blythii*).

Families	Host Range	Genome Type	Overall (%)	Sample Type (%)			Geographical Location Group (%)		Landscape Type (%)		Bat Species (%)						
				S	F	G	M	C	N	H	RF	MS	MM	ME	MB	ES	Undetermined/Mixed
*Iflaviridae*	Invertebrates	(+)ssRNA	44.663	0.058	81.424	9.780	57.391	5.951	2.385	76.786	85.236	0.061	0.000	0.424	0.000	0.000	8.364
*Siphoviridae*	Bacteria or archaea	dsDNA	9.591	12.849	2.381	18.418	10.599	6.524	20.755	1.109	1.233	19.523	7.435	5.932	27.895	12.832	17.775
*Podoviridae*	Bacteria or archaea	dsDNA	7.352	7.828	3.198	12.944	7.971	5.470	12.604	3.361	1.884	9.364	3.770	2.119	24.211	8.407	13.224
*Parvoviridae*	Vertebrates, Unclassified or unknown, Invertebrates	ssDNA	5.996	11.810	0.125	12.040	7.920	0.143	7.412	4.920	3.216	0.000	0.105	0.000	0.000	0.000	10.446
*Myoviridae*	Bacteria or archaea	dsDNA	4.048	9.021	2.667	4.166	2.901	7.536	8.702	0.512	0.488	12.179	10.785	15.678	8.684	5.752	6.597
*Retroviridae*	Vertebrates, Invertebrates	ssRNA-RT	2.333	14.464	0.526	0.452	0.995	6.401	4.442	0.730	1.821	11.016	21.675	26.271	10.263	3.761	0.416
*Mimiviridae*	Protozoa, Unclassified or unknown	dsDNA	1.728	7.540	1.178	0.390	0.635	5.051	3.475	0.401	0.624	9.058	9.634	8.475	3.421	2.434	1.666
*Ackermannviridae*	Bacteria or archaea	dsDNA	1.477	6.040	0.396	1.329	0.054	5.808	2.514	0.690	0.687	3.611	10.471	0.424	0.263	6.858	1.553
*Iridoviridae*	Vertebrates, Invertebrates	dsDNA	1.126	5.905	0.326	0.508	0.561	2.843	2.156	0.343	0.818	5.263	6.911	13.136	5.263	1.106	0.482
*Poxviridae*	Vertebrates, Invertebrates	dsDNA	1.017	3.405	0.762	0.508	0.205	3.487	2.150	0.156	0.273	3.488	5.131	0.847	2.368	29.425	0.595
*Phycodnaviridae*	Plants or algae	dsDNA	0.878	3.443	0.652	0.264	0.286	2.679	1.799	0.178	0.310	4.162	4.188	2.542	1.842	2.655	0.922
*Microviridae*	Bacteria or archaea	ssDNA	0.620	0.385	0.296	1.155	0.740	0.256	1.025	0.312	0.178	0.612	0.628	0.000	0.263	0.221	1.148
*Reoviridae*	Vertebrates, Invertebrates	dsRNA	0.615	0.519	0.030	1.461	0.000	2.485	1.371	0.040	0.010	0.122	2.094	0.000	0.000	1.991	1.249
*Permutotetraviridae*	Unclassified or unknown, Invertebrates	(+)ssRNA	0.531	0.000	1.033	0.028	0.703	0.010	0.000	0.935	1.081	0.000	0.000	0.000	0.000	0.000	0.024
*Inoviridae*	Bacteria or archaea	ssDNA	0.443	0.692	0.015	0.946	0.545	0.133	0.949	0.058	0.000	0.796	1.047	5.508	0.000	0.221	0.821
*Herpesviridae*	Vertebrates	dsDNA	0.369	1.808	0.236	0.035	0.128	1.104	0.820	0.027	0.073	2.999	2.618	0.847	0.000	1.327	0.297
*Picobirnaviridae*	Vertebrates, Unclassified or unknown	dsRNA	0.306	0.000	0.000	0.842	0.000	1.237	0.697	0.009	0.000	0.000	0.000	0.000	0.000	0.000	0.720
*Circoviridae*	Vertebrates, Unclassified or unknown, Fungi, Invertebrates	ssDNA	0.293	0.000	0.000	0.807	0.387	0.010	0.680	0.000	0.000	0.000	0.000	0.000	0.000	0.000	0.690
*Flaviviridae*	Vertebrates	(+)ssRNA	0.273	1.866	0.020	0.049	0.027	1.022	0.551	0.062	0.220	1.958	1.780	0.000	0.000	2.212	0.042
*Nodaviridae*	Vertebrates, Unclassified or unknown, Invertebrates	(+)ssRNA	0.202	0.135	0.000	0.508	0.013	0.777	0.258	0.160	0.031	0.061	0.000	0.000	0.000	0.000	0.434
*Dicistroviridae*	Invertebrates	(+)ssRNA	0.202	0.038	0.000	0.543	0.003	0.808	0.205	0.200	0.010	0.000	0.000	0.000	0.000	0.000	0.464
*Partitiviridae*	Plants or algae	dsRNA	0.190	0.000	0.165	0.292	0.141	0.337	0.182	0.196	0.173	0.000	0.000	0.000	0.000	0.000	0.250
*Totiviridae*	Protozoa, Invertebrates	dsRNA	0.187	0.000	0.341	0.042	0.229	0.061	0.035	0.303	0.000	0.000	0.000	0.000	0.000	0.000	0.440
*Astroviridae*	Vertebrates	(+)ssRNA	0.175	0.327	0.000	0.362	0.003	0.695	0.398	0.004	0.000	0.979	0.105	0.000	0.000	0.000	0.309
*Coronaviridae*	Vertebrates	(+)ssRNA	0.154	0.038	0.000	0.410	0.010	0.593	0.053	0.232	0.010	0.000	0.000	0.000	0.000	0.000	0.351
*Picornaviridae*	Vertebrates	(+)ssRNA	0.134	0.000	0.000	0.369	0.003	0.532	0.211	0.076	0.000	0.000	0.000	0.000	0.000	0.000	0.315
*Solemoviridae*	Plants or algae	(+)ssRNA	0.114	0.000	0.005	0.306	0.000	0.460	0.012	0.191	0.000	0.000	0.000	0.000	0.000	0.221	0.262
*Polydnaviridae*	Invertebrates	dsDNA	0.089	0.654	0.000	0.007	0.064	0.164	0.152	0.040	0.026	0.367	1.257	3.390	0.789	0.000	0.006
*Tymoviridae*	Plants or algae	(+)ssRNA	0.073	0.000	0.000	0.202	0.000	0.297	0.170	0.000	0.000	0.000	0.000	0.000	0.000	0.000	0.173
*Pithoviridae*	Protozoa, Unclassified or unknown	dsDNA	0.053	0.192	0.045	0.014	0.013	0.174	0.105	0.013	0.010	0.184	0.419	0.424	0.000	0.000	0.065
*Caulimoviridae*	Plants or algae	dsDNA-RT	0.051	0.308	0.005	0.021	0.040	0.082	0.105	0.009	0.021	0.428	0.000	0.000	0.263	1.106	0.018
*Virgaviridae*	Plants or algae	(+)ssRNA	0.048	0.000	0.000	0.132	0.000	0.194	0.111	0.000	0.000	0.000	0.000	0.000	0.000	0.000	0.113
*Baculoviridae*	Invertebrates	dsDNA	0.043	0.231	0.005	0.028	0.010	0.143	0.088	0.009	0.016	0.428	0.209	0.000	0.000	0.000	0.030
*Adenoviridae*	Vertebrates, Unclassified or unknown	dsDNA	0.040	0.077	0.060	0.000	0.020	0.102	0.076	0.013	0.016	0.061	0.000	0.000	0.000	0.000	0.071
*Metaviridae*	Invertebrates	ssRNA-RT	0.035	0.212	0.005	0.014	0.034	0.041	0.082	0.000	0.010	0.428	0.000	0.000	0.263	0.442	0.012
*Ascoviridae*	Invertebrates	dsDNA	0.030	0.115	0.020	0.014	0.010	0.092	0.064	0.004	0.005	0.184	0.209	0.000	0.000	0.000	0.036
*Luteoviridae*	Plants or algae	(+)ssRNA	0.025	0.000	0.010	0.056	0.000	0.102	0.018	0.031	0.000	0.000	0.000	0.000	0.000	0.442	0.048
*Tombusviridae*	Plants or algae	(+)ssRNA	0.025	0.000	0.000	0.070	0.000	0.102	0.006	0.040	0.000	0.000	0.000	0.000	0.000	0.000	0.059
*Alphatetraviridae*	Invertebrates	(+)ssRNA	0.023	0.019	0.000	0.056	0.003	0.082	0.041	0.009	0.005	0.000	0.000	0.000	0.000	0.000	0.048
*Peribunyaviridae*	Different eucaryonts, Vertebrates	(-)ssRNA	0.020	0.000	0.000	0.056	0.010	0.051	0.029	0.013	0.000	0.000	0.000	0.000	0.000	0.000	0.048
*Polycipiviridae*	Invertebrates	(+)ssRNA	0.020	0.000	0.000	0.056	0.020	0.020	0.023	0.018	0.000	0.000	0.000	0.000	0.000	0.000	0.048
*Bromoviridae*	Plants or algae	(+)ssRNA	0.015	0.000	0.000	0.042	0.000	0.061	0.035	0.000	0.000	0.000	0.000	0.000	0.000	0.000	0.036
*Phenuiviridae*	Vertebrates, Unclassified or unknown, Invertebrates	(-)ssRNA	0.015	0.000	0.000	0.042	0.000	0.061	0.035	0.000	0.000	0.000	0.000	0.000	0.000	0.000	0.036
*Alloherpesviridae*	Vertebrates	dsDNA	0.015	0.096	0.000	0.007	0.000	0.061	0.029	0.004	0.026	0.000	0.000	0.000	0.000	0.000	0.006
*Leviviridae*	Bacteria or archaea	(+)ssRNA	0.015	0.019	0.000	0.035	0.000	0.061	0.035	0.000	0.005	0.000	0.000	0.000	0.000	0.000	0.030
*Marseilleviridae*	Protozoa, Unclassified or unknown	dsDNA	0.013	0.077	0.005	0.000	0.003	0.041	0.018	0.009	0.005	0.061	0.209	0.424	0.000	0.000	0.000
*Nudiviridae*	Invertebrates	dsDNA	0.013	0.019	0.005	0.021	0.003	0.041	0.023	0.004	0.005	0.000	0.000	0.000	0.000	0.000	0.024
*Rhabdoviridae*	Invertebrates, Plants or algae, Vertebrates, Unclassified or unknown	(-)ssRNA	0.013	0.000	0.000	0.035	0.000	0.051	0.029	0.000	0.000	0.000	0.000	0.000	0.000	0.000	0.030
*Genomoviridae*	Unclassified or unknown	ssDNA	0.010	0.019	0.000	0.021	0.010	0.010	0.012	0.009	0.005	0.000	0.000	0.000	0.000	0.000	0.018
*Potyviridae*	Plants or algae	(+)ssRNA	0.008	0.058	0.000	0.000	0.003	0.020	0.012	0.004	0.005	0.000	0.105	0.424	0.000	0.000	0.000
*Birnaviridae*	Vertebrates, Invertebrates	dsRNA	0.008	0.000	0.000	0.021	0.000	0.031	0.018	0.000	0.000	0.000	0.000	0.000	0.000	0.000	0.018
*Malacoherpesviridae*	Invertebrates	dsDNA	0.008	0.058	0.000	0.000	0.007	0.010	0.012	0.004	0.000	0.061	0.105	0.424	0.000	0.000	0.000
*Secoviridae*	Plants or algae	(+)ssRNA	0.005	0.000	0.000	0.014	0.003	0.010	0.012	0.000	0.000	0.000	0.000	0.000	0.000	0.000	0.012
*Hepeviridae*	Vertebrates	(+)ssRNA	0.005	0.019	0.000	0.007	0.000	0.020	0.006	0.004	0.000	0.061	0.000	0.000	0.000	0.000	0.006
*Betaflexiviridae*	Plants or algae	(+)ssRNA	0.005	0.019	0.000	0.007	0.000	0.020	0.012	0.000	0.005	0.000	0.000	0.000	0.000	0.000	0.006
*Nanoviridae*	Plants or algae	dsDNA	0.003	0.000	0.000	0.007	0.003	0.000	0.006	0.000	0.000	0.000	0.000	0.000	0.000	0.000	0.006
*Spiraviridae*	Unclassified or unknown	ssDNA	0.003	0.000	0.000	0.007	0.000	0.010	0.006	0.000	0.000	0.000	0.000	0.000	0.000	0.000	0.006
*Caliciviridae*	Vertebrates	(+)ssRNA	0.003	0.000	0.000	0.007	0.000	0.010	0.006	0.000	0.000	0.000	0.000	0.000	0.000	0.000	0.006
*Bicaudaviridae*	Bacteria or archaea	dsDNA	0.003	0.000	0.005	0.000	0.000	0.010	0.006	0.000	0.000	0.000	0.000	0.000	0.000	0.000	0.006
*Autolykiviridae*	Unclassified or unknown	dsDNA	0.003	0.000	0.000	0.007	0.003	0.000	0.000	0.004	0.000	0.000	0.000	0.000	0.000	0.000	0.006
*Bornaviridae*	Vertebrates	(-)ssRNA	0.003	0.019	0.000	0.000	0.003	0.000	0.006	0.000	0.000	0.061	0.000	0.000	0.000	0.000	0.000
*Polyomaviridae*	Vertebrates	dsDNA	0.003	0.000	0.000	0.007	0.003	0.000	0.006	0.000	0.000	0.000	0.000	0.000	0.000	0.000	0.006
*Asfarviridae*	Vertebrates	dsDNA	0.003	0.019	0.000	0.000	0.003	0.000	0.006	0.000	0.000	0.061	0.000	0.000	0.000	0.000	0.000
Unclassified or unknown	Bacteria or archaea, Vertebrates, Different eucaryonts, Plants or algae, Unclassified or unknown, Invertebrates, Protozoa	(-)ssRNA, Unclassified or unknown RNA, ssDNA, (+)ssRNA, dsDNA, dsRNA, Unclassified or unknown	14.238	9.598	4.060	30.041	7.278	35.409	22.759	7.765	1.453	12.362	9.110	12.712	14.211	18.584	29.114

**Table 3:** Metadata summary of the sequenced pools. Table lists geographical locations (1–11), location groups (M—Mediterranean, C—continental), sample types (S—oral swab, F—feces, G—guano), types of landscape (N—natural, H—with human activity, anthropogenic), bat species examined (MS—*Miniopterus schreibersii*, MM—*Myotis myotis*, RF—*Rhinolophus ferrumequinum*, ME—*Myotis emarginatus*, ES—*Eptesicus serotinus*, MB—*Myotis blythii*), with total numbers of sequences, the numbers of viral sequences, and the zeroth- and first-order Hill diversity indices (^0,1^D) based on taxonomy and host range.

No.	Landscape Type	Sample Type	Location	Location Group	Species	All Sequences	Viral Sequences (%)	^0^D_Tax. Family_	^1^D_Tax. Family_	^0^D_Host Range_	^1^D_Host Range_
1	N	F	1	C	MS	7986	645 (8.077)	22	0.210	7	0.335
2	N	S	1	C	MS	76,457	170 (0.222)	15	0.090	6	0.307
3	N	S	1	C	RF	107,464	210 (0.195)	17	0.109	6	0.218
4	N	S	9	M	RF	9988	747 (7.479)	15	0.435	5	0.574
5	N	S	9	M	MS	2560	35 (1.367)	7	0.192	5	0.414
6	N	S	9	M	MB	16,903	159 (0.941)	12	0.138	6	0.314
7	H	G	2	C	/	78,222	1973 (2.522)	35	0.255	7	0.315
8	H	S	2	C	MM	108,367	156 (0.144)	16	0.112	6	0.280
9	N	F	4	C	RF	58,257	170 (0.292)	12	0.292	6	0.603
10	N	F	4	C	ES	10,238	320 (3.126)	17	0.158	6	0.269
11	N	F	4	C	MS	83,747	340 (0.406)	20	0.154	7	0.236
12	N	S	4	C	RF	48,398	169 (0.349)	12	0.152	6	0.320
13	N	S	4	C	RF	39,251	151 (0.385)	14	0.134	7	0.262
14	N	S	4	C	MS	42,389	111 (0.262)	17	0.076	7	0.248
15	N	S	4	C	RF	79,074	256 (0.324)	20	0.093	7	0.253
16	N	S	4	C	ES	69,349	132 (0.19)	15	0.081	7	0.227
17	N	F	11	M	MS	4168	15 (0.36)	7	0.159	5	0.276
18	N	F	11	M	MS	6120	15 (0.245)	8	0.137	3	0.364
19	N	G	11	M	/	23,648	1806 (7.637)	23	0.214	7	0.435
20	N	S	11	M	MS, MN	39,015	157 (0.402)	17	0.097	6	0.261
21	N	S	11	M	MM	40,471	262 (0.647)	15	0.108	7	0.248
22	N	S	11	M	MS	7833	183 (2.336)	10	0.194	5	0.440
23	N	S	11	M	RF	10,063	129 (1.282)	14	0.145	6	0.362
24	H	F	7	M	RF	29,564	16,998 (57.496)	7	0.779	4	0.857
25	H	G	7	M	/	10,662	2644 (24.798)	21	0.232	7	0.472
26	H	S	7	M	RF	38,090	114 (0.299)	8	0.272	5	0.517
27	H	S	7	M	ME	35,720	236 (0.661)	18	0.109	6	0.247
28	N	S	8	M	MB	2860	13 (0.455)	8	0.136	4	0.336
29	N	S	8	M	MS	13,045	196 (1.502)	13	0.174	6	0.416
30	N	F	3	C	MS	43,707	2129 (4.871)	17	0.140	7	0.345
31	N	G	3	C	/	42,904	1853 (4.319)	40	0.175	7	0.206
32	N	S	3	C	MS	42,697	277 (0.649)	19	0.095	7	0.230
33	N	S	3	C	MM	55,162	302 (0.547)	20	0.102	7	0.235
34	N	S	3	C	RF	63,273	235 (0.371)	16	0.106	6	0.267
35	N	S	3	C	MS	53,987	101 (0.187)	14	0.125	5	0.285
36	N	S	3	C	MM	26,439	80 (0.303)	14	0.130	5	0.333
37	N	S	10	M	MB	16,549	208 (1.257)	11	0.156	7	0.351
38	N	S	10	M	MS	7997	160 (2.001)	12	0.180	6	0.414
39	H	F	5	M	MS, RF	14,731	267 (1.813)	14	0.121	6	0.202
40	H	F	5	M	MS, RF	4915	37 (0.753)	9	0.165	6	0.326
41	H	S	5	M	RF	3220	36 (1.118)	9	0.157	4	0.365
42	N	G	6	M	/	39,053	5116 (13.1)	9	0.181	7	0.355
43	N	S	6	M	MS	51,000	214 (0.42)	22	0.082	7	0.219

## Supplementary Materials

The following are available online at https://www.mdpi.com/1999-4915/12/8/891/s1, Table S1: Viral metagenomics data in detail.
